# Assessing environmental and radiological impacts and lithological mapping of beryl-bearing rocks in Egypt using high-resolution sentinel-2 remote sensing images

**DOI:** 10.1038/s41598-023-38298-0

**Published:** 2023-07-17

**Authors:** Farrage M. Khaleal, Mohammed Z. El-Bialy, Gehad M. Saleh, El Saeed R. Lasheen, Mohamed S. Kamar, Mohamed M. Omar, Mohamed N. El-dawy, Ahmed Abdelaal

**Affiliations:** 1 Nuclear Materials Authority, P.O. Box 530, El Maadi, Cairo, Egypt; 2 Geology Department, Faculty of Science, Port Said University, Port Said, Egypt; 3 Geology Department, Faculty of Science, Al-Azhar University, P.O. Box 11884, Cairo, Egypt; 4 Environmental Sciences Department, Faculty of Science, Port Said University, Port Said, 42522 Egypt

**Keywords:** Environmental sciences, Solid Earth sciences

## Abstract

Emerald and other beryls represent a family of the most valuable gemstone around the world and particularly in Egypt. Beryllium (Be) contents in beryl-bearing bedrocks in south Sinai (Wadi Ghazala and Wadi Sedri), and in central and south Eastern Desert of Egypt (Igla area, Zabara-Um Addebaa belt, Homret Akarem, and Homret Mukpid) were investigated in this study. The environmental risk levels of Be, associated major ions, and heavy metals in groundwater nearby to beryl-bearing mineralization were also evaluated. Results showed that Be contents ranged from 1 to 374 ppm in beryl-bearing bedrocks, while in nearby groundwater, Be content has a range of 0.0001–0.00044 mg/L with an average of 0.00032 mg/L, which is within the permissible levels and below (0.004) the U.S. EPA maximum contaminant level (MCL). Most levels of heavy metals (e.g., Be, B, Ni, V, Fe, and Al) in the investigated groundwater of central and south Eastern Desert and south Sinai are within the permissible levels and below their corresponding U.S. EPA MCLs. This study also investigated the radiological risk of natural radionuclides distributed in beryl-bearing bedrocks in the study area using gamma spectrometry; Sodium Iodide [NaI(Tl)] scintillation detector. Among the estimated mean ^238^U, ^232^Th, and ^226^Ra activity concentrations of the studied beryl-bearing rocks, Homret Mukpid (79, 87.15, 60.26 Bq kg^−1^) and Homret Akarem (111.6, 51.17, 85.1 Bq kg^−1^) contain the highest values. This may be attributed to their highly fractionated granitic rocks that host uranium and thorium reservoir minerals such as zircon, allanite, and monazite. The estimated data of multi-radiological parameters such as absorbed gamma dose, outdoor and indoor annual effective dose, radium equivalent activity, internal and external indices, index of excess cancer, and effective dose to human organs reflecting no significant impacts from the emitted natural gamma radiation.

## Introduction

Emerald and beryl mineralization in Egypt are restricted to south Sinai (Wadi (W.) Sedri and W. Ghazala), central Eastern Desert (CED) (Igla area), and south Eastern Desert (SED) (Zabara-Um Addebaa belt, Homret Akarem and Homret Mukpid areas)^1–7^. Green emerald is recorded in Zabara-Um Addebaa belt^6,8^. This belt comprises to the north, Um Addebaa, Um Kabu, Um El Kheran, Um Solimat, Nugrus, Abu Rusheid, Sikait, and Zabara. Beryl is the main source of beryllium (Be) that is distinguished by highly significant attributes such as high thermal and electrical conductivity and can be used in nuclear and military industries^9^. Beryl, emerald, and aquamarine are the main types that are characterized by colorless, green, and blue color as a result of chromophore elements^10–12^. These deposits are relatively rare and form in a restricted geological setting. They may be ascribed to granites and their linked rocks^12–15^ and/or during regional metamorphism^12–15^.

Radionuclides concentrations are widely distributed in various rocks (certainly granitic rocks), soil, and sediments^16–18^. ^238^U, ^232^Th, ^226^Ra, and ^40^K represent the dominant terrestrial radionuclides of gamma radiation. These radionuclides are abundant within crustal rocks certainly the highly evolved granitic rocks and pegmatites. Many diseases may be produced from long time exposure and inhalation of radon gas, which is the product of radium such as acute leucopenia and bone cancers^16,19–21^.

Beryllium is used in various industries to make nuclear weapons and reactors, aircraft and space vehicle structures, and X-ray machines, thus the entry of Be into the environment threatens the sustainability of the environment including surface water, groundwater, and human health as well^22^. Be is potentially a highly toxic element that may be mobilized especially under acidic conditions in natural waters^23^. Seepage or percolation of Be-contaminated groundwater may result in the accumulation of Be in soil and highly threaten the soil quality and may pose human health risks^22^. Numerous studies in literature investigated Be concentrations in emerald and other beryl-bearing rocks, while few studies worldwide focused on investigating Be levels in groundwater derived from or nearby beryl-bearing bedrocks.

Several recent studies are applying the remote sensing multispectral images such as the combination of Landsat-8 OLI, ALOS/PALSAR, and SRTM data which effectively display the structural and lithological mapping of the rock units in CED and SED of Egypt that were previously difficult to reveal by optical images^24,25^. Other studies are applying combination of Landsat-8 OLI and ASTER datasets for lithological mapping of the rock units in CED^25^, SED^26,27^, and also in south Sinai, Egypt^28^. Moreover, Sentinel-2A satellite data contains higher spectral (13 bands) and widespread spatial resolution (10–60 m) in the VNIR to SWIR range than common multispectral data, such as OLI and ASTER data^29,30^. Accordingly, Sentinel-2A images were used for better interpretation and enhancing the lithological mapping tools of the beryl-bearing bedrocks in this study, compared to the classified lithological rocks units obtained from the typical geological maps.

The objectives of this study are; (1) mapping and verifying the lithology of beryl-bearing bedrock occurrences using high-resolution remote sensing imagery (e.g., Sentinel-2A); (2) assessing the Be concentrations in beryl-bearing bedrocks; (3) assessing Be concentrations for the first time in this study, along with associated major dissolved ions and heavy metals in nearby groundwater in south Sinai and south Eastern Desert of Egypt, to evaluate the environmental risk of Be and associated metals, and their adverse impacts on shallow groundwater quality, the environment sustainability, and human health; and (4) investigating the distribution of natural radionuclides in emerald and other beryl-bearing bedrocks in six representative areas; Igla area in CED, Zabara-Um Addebaa belt, Homret Akarem, and Homret Mukpid in SED, and W. Ghazala, and W. Sedri in south Sinai, Egypt, to evaluate their radiological risk on the environment and human health.

## Geologic setting

**Figure 1:**
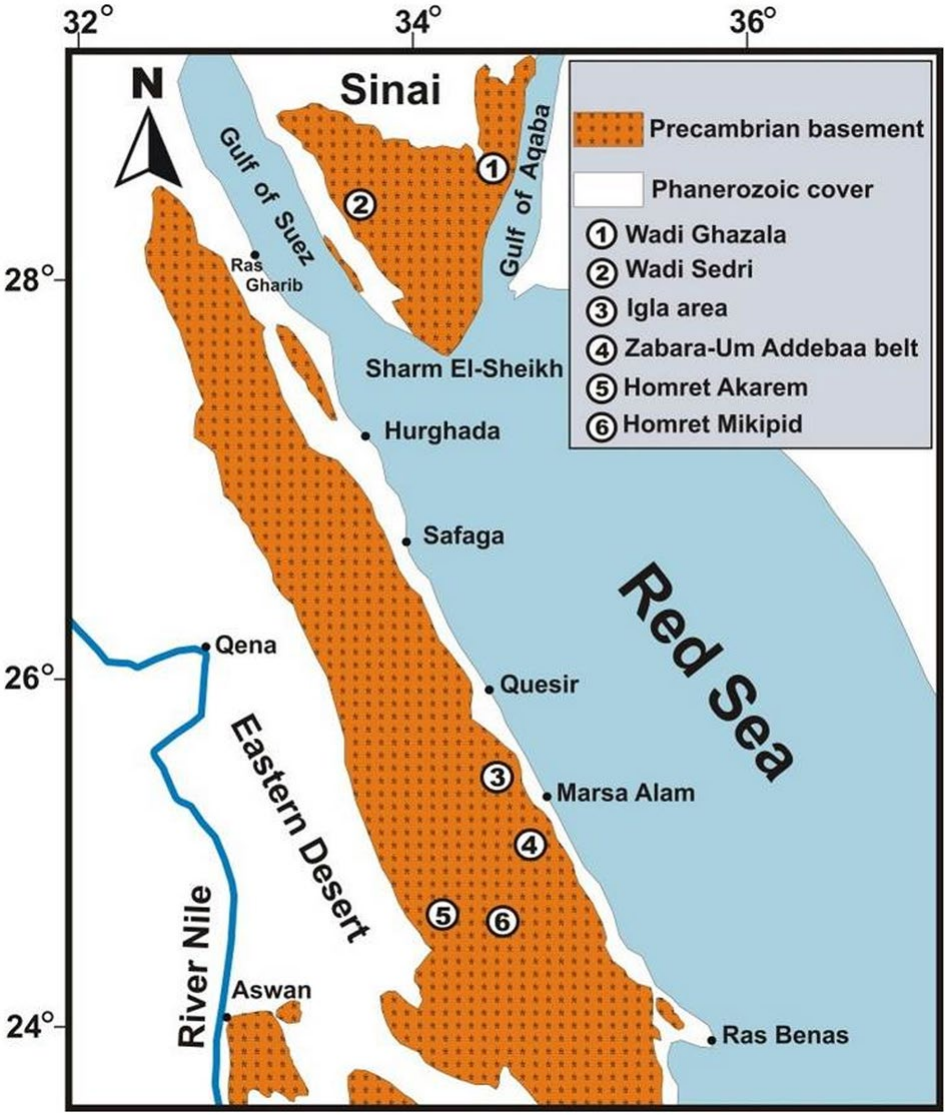
Location map for the basement rocks of Neoproterozoic age as a part of the Arabina Nubian Shield (ANS), showing occurrences of beryls mineralization in Egypt. This figure has been drawn by using Coreldraw program version 2012.

Emerald and other beryl mineralization occur in south Sinai (W. Ghazala^3^, and W. Sedri)^7^. They are also recoded in Igla area in CED of Egypt^1^, and in Zabara- Um Addebaa belt, Homret Mukpid and Homret Akarem areas in SED of Egypt^2,4,5,12^, (Fig. 1).

### Sinai occurrences

#### Wadi Ghazala

**Figure 2:**
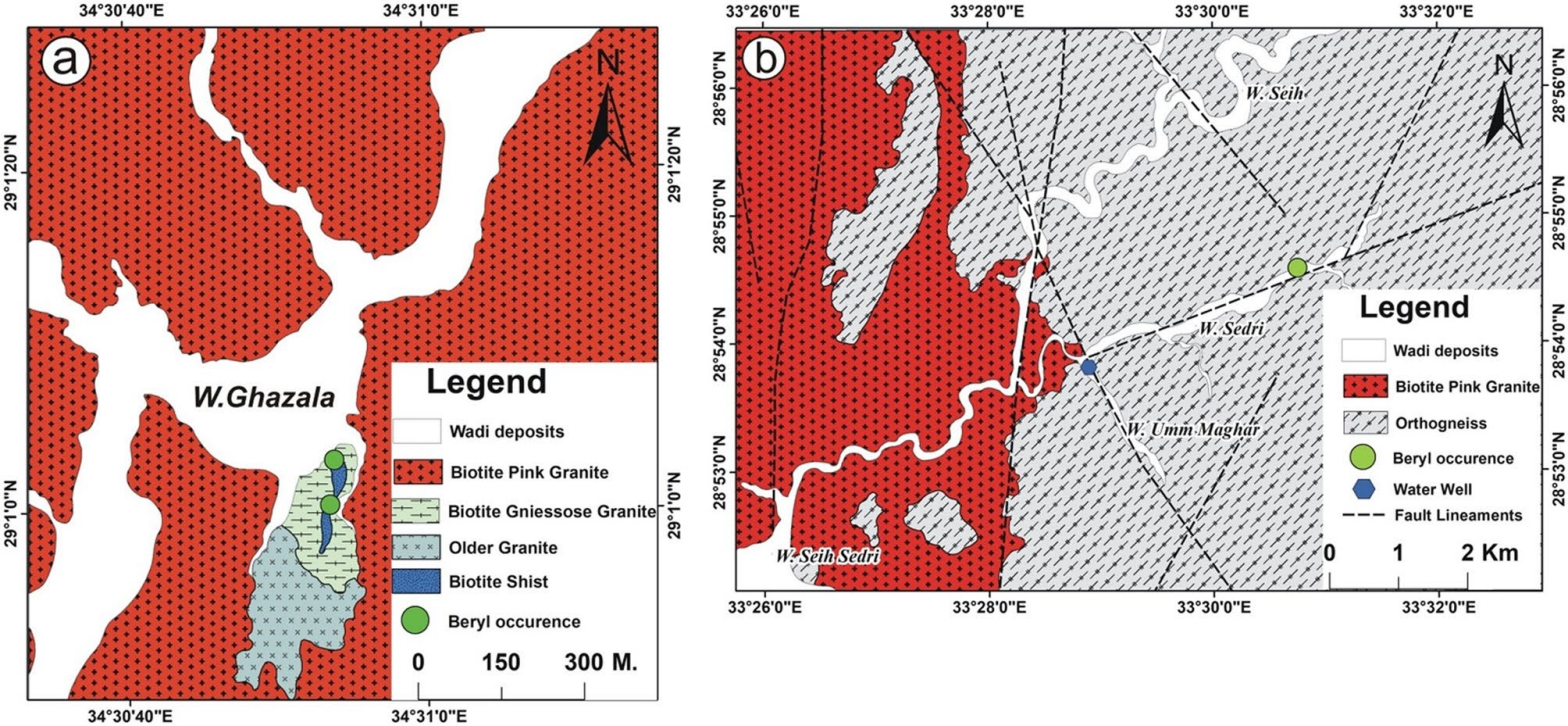
Detailed geological maps of (**a**) Wadi Ghazala area, southeastern Sinai, Egypt^3^, and (**b**) Wadi Sedri area, southwestern Sinai, Egypt^7^. This figure has been drawn by using Coreldraw program version 2012.

**Figure 3:**
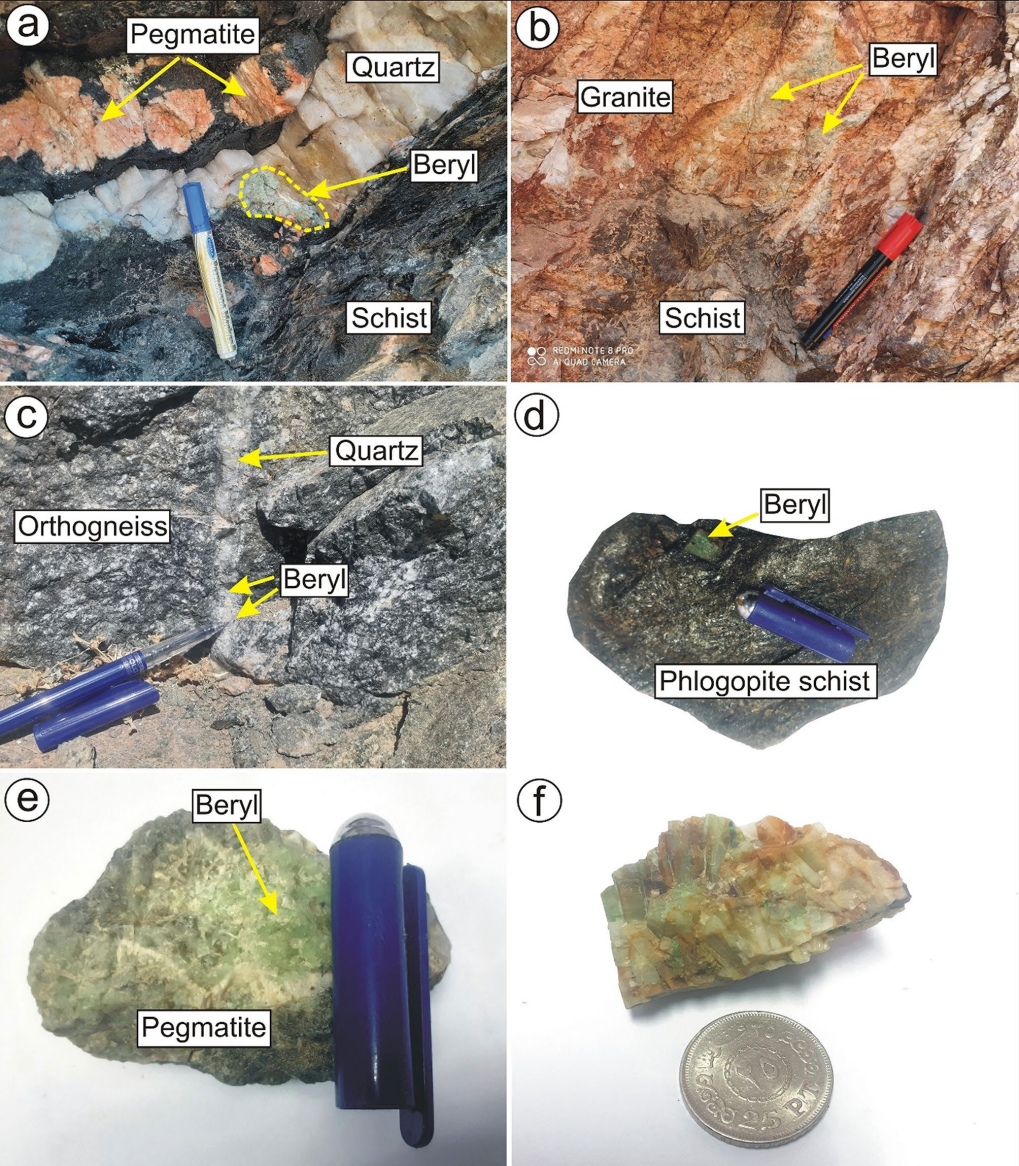
Field photographs showing beryl mineralization hosted in; (**a**) quartz veins and (**b**) biotite gneissose granites in W. Ghazala, southeastern Sinai, Egypt; (**c**) dissiminated in quartz veins within orthogenesis in Wad. Sedri, southwestern Sinai, Egypt; (**d**) phlogopite schist and (**e**) pegmatite in Zabara-Um Addebaa belt, SED of Egypt; and (**f**) pale green beryl crystals from Homret Akarem area, SED of Egypt.

W. Ghazala locates about 25 km west of Nuweiba, southeastern Sinai (Fig. 2a), and is dominated by older granitoid, biotite gneissose granites, and biotite pink granites^3^. Biotite pink granites are the dominant rocks in W. Ghazala area, which are sheared and intruded by many basic dykes. Beryl mineralization in W. Ghazala area is associated with three rock types: pegmatite, quartz veins (Fig. 3a), and biotite gneissose granites (Fig. 3b).

#### Wadi Sedri

W. Sedri is located to the north of W. Feiran, southwestern Sinai, and mainly covered by orthogneiss and biotite pink granites (Fig. 2b). Orthogneiss rocks are well exposed in W. Um Maghar, W. Sedri and W. Seih-Sedri areas^7^. The biotite pink granites intruded sharply with orthogneiss and showing high relief with coarse-grained and red color (Fig. 2b). Beryl-bearing quartz veins are cross-cutting orthogneiss rocks (Fig. 3c).

### Eastern Desert occurrences

#### Igla area

**Figure 4:**
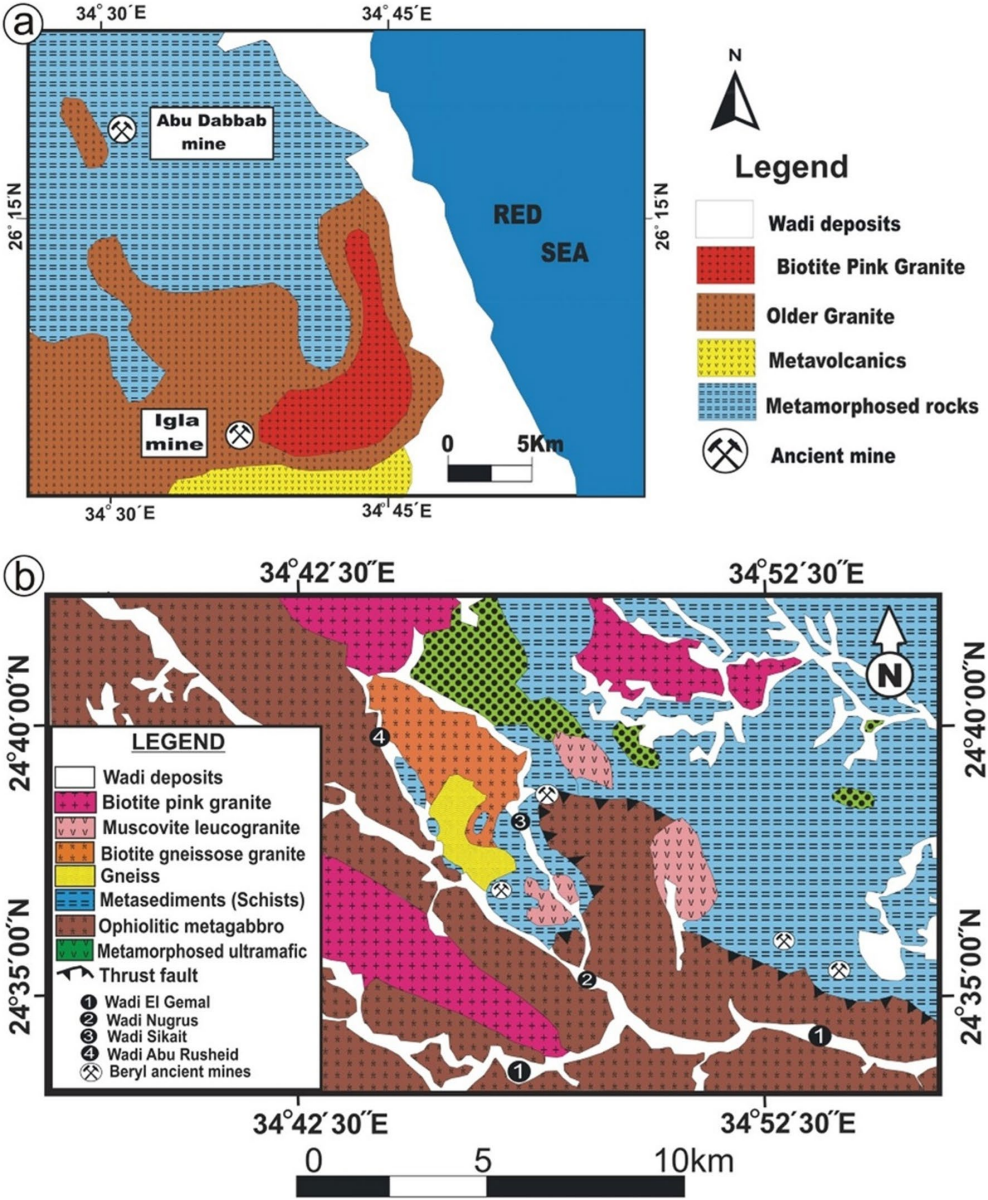
Dtailed geological maps of (**a**) Igla mine area, CED of Egypt^31,32^, and (**b**) Zabara-Um Addebaa belt, SED of Egypt^12^. This figure has been drawn by using Coreldraw program version 2012.

Igla mine area locates in the CED of Egypt (Fig. 4a) and is dominated by metavolcanics, older granites, and biotite pink granites^31,32^. The metavolcanics rocks are well exposed in the southern part of the study area (Fig. 4a). Biotite pink granites are pink to red, isolated plutons, and reveal sharp contacts with surrounding rocks that characterized by coarse-grained crystals and dissected by numerous dykes. Igla area has common mineralization such as malachite, albite, cassiterite, zinnwaldite and beryl.

#### Zabara-Um Addebaa belt

Zabara-Um Addebaa belt locates in the SED of Egypt (Fig. 4b). The belt extends to 33 km long and 9 km wide including areas of Um Addebaa, Um Kabu, Um El Kheran, Um Solimat, Nugrus, Abu Rusheid, Sikait, and Zabara. The belt is dominated by ophiolitic rocks (ultramafic and mafic rocks embedded in mélange matrix), gneiss rocks, biotite gneissose granites, muscovite leucogranites, biotite pink granites and post granitic dykes and veins. Ophiolitic rocks in Egypt are commonly dismembered with variable tectonic regimes^33–37^. The ultramafic rocks are highly tectonized forming fold-thrust sheets over ophiolitic metagabbros (ENE-WSW)^38,39^. Ophiolitic mélange includes many varieties of schistose rocks such as graphite-biotite schist, garnet-hornblende-biotite schist, quartzo-feldspathic schist, and phlogopite schist, which contains the beryl mineralization^35^. The contact between the gneisses and schist rocks is usually sharp and variable mineralization such as radioactive minerals and columbite. Biotite gneissose granites form high terrain in the central northeast of the Abu Rusheid pluton^33^. The biotite pink granites are characterized by pink color and medium grain size and with equigranular texture. They are composed mainly of potash feldspar, plagioclase, and quartz associated with biotite as the main mafic mineral. Beryl mineralization in Zabara-Um Addebaa belt occurs in either phlogopite schist (Fig. 3d) or quartz and pegmatite veins (Fig. 3e).

#### Homret Akarem

**Figure 5:**
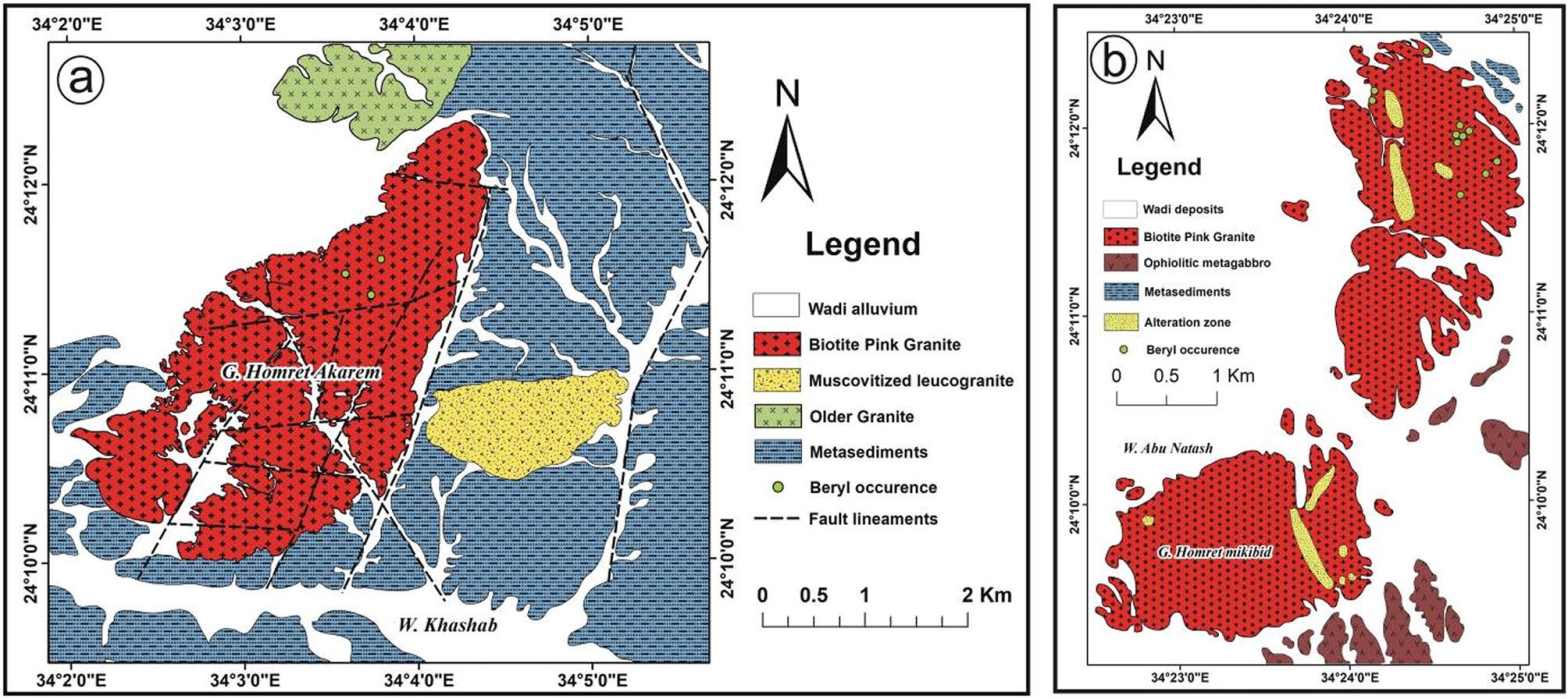
Geologic maps of: (**a**) Homret Akarem area, SED, Egypt^5,40^; and (**b**) Homret Mukpid area, SED, Egypt^5,40^. This figure has been drawn by using Coreldrow program version 2012.

Homret Akarem area lies in the SED of Egypt in an area ~ 40 km^2^ (Fig. 5a) and is covered by metasediments, older granites, muscovite leucogranites, and biotite pink granites. Metasediments represent most of the mapped area and are intruded by the surrounding granitic rocks. Older granites are greyish with low relief, very weathered and fractured. Muscovite leucogranites intrude metasediments and can be distinguished by their white color. Biotite pink granites show an elongated mass with NE-SW direction. They are medium to coarse-grained and affected by extensive alteration. Beryl mineralization in Homret Akarem area occurs within pegmatite veins cutting across the biotite pink granites with pale green color (Fig. 3f)^6,36^.

#### Homret Mukpid

Homret Mukpid area lies in the SED of Egypt with an area of ~ 24 Km^2^ (Fig. 5b) and is comprised of metasediments, metagabbros, and highly fractured and jointed biotite pink granites^5,36^. Metasediments are well exposed in the northern part of the study area characterized by grayish black to greenish color. Metagabbros are greenish to pale green color with medium relief. Beryl mineralization is disseminated in granitic bodies with blue color (Fig. 5b).

## Materials and methods

### Lithological mapping of beryl-bearing bedrocks

**Figure 6:**
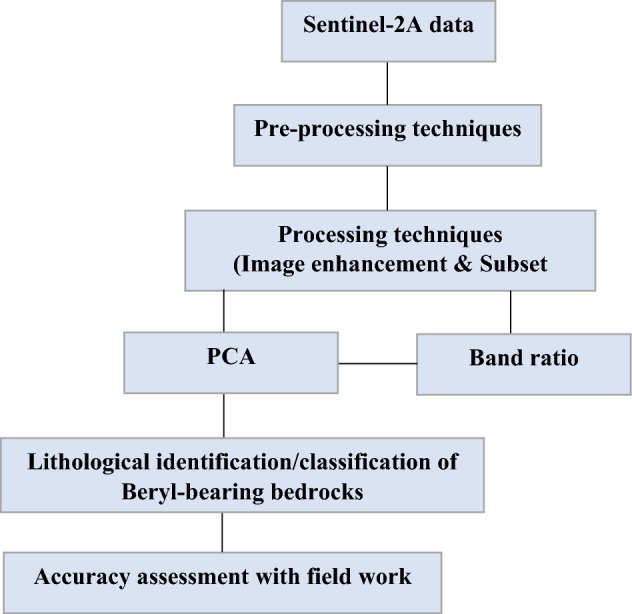
Flowchart summarized the lithological classification methodology used in the present study.

In this study, together with geological maps, an approach to facilitate the geological interpretation of Sentinel-2A high-resolution images (scenes: 8; source: U.S. Geological Survey (USGS), website: (https://earthexplorer.usgs.gov/), were used for providing a greater improvement in the geological features in hyper-arid areas at Sinai and South Eastern Desert of Egypt. The Sentinel-2A image has 13 spectral bands in the VNIR and SWIR spectral range, with four bands at 10 m, six bands at 20 m, and three atmospheric correction bands at 60 m^25^ as shown in Table 1. The cloud-free Sentinel-2A images were automatically atmospherically corrected and orthorectified using the Sentinel Application Platform (SNAP) software as summarized in Fig. 6. These approaches used histogram treatments, image enhancement, and subset tools, and all Sentinel-2A VNIR bands were re-sampled to (10 × 10 m) pixel size (Fig. 6). Band ratios were applied for better lithological discrimination^25^ (Fig. 6). A false-color (RGB) composite contains a large volume of information, including topographic information and geological roughness. Therefore, Principal Component Analysis (PCA) was applied to improve the visual interpretation to reveal the lithological mapping enhancement of the beryl-bearing bedrocks in the study area. The Sentinel-2A PCA output images were mosaiced and exported in ArcGIS 10.6 software.

**Table 1:** Description of the spectral bands of Sentinel-2A data.

Band	Band description	Central wavelength (µm)	Spatial resolution (m)
1	Coastal aerosol	0.443	60
2	Blue	0.490	60
3	Green	0.560	10
4	Red	0.665	10
5	Vegetation Red Edge	0.705	10
6	Vegetation Red Edge	0.740	20
7	Vegetation Red Edge	0.783	20
8	NIR	0.842	10
8A	Vegetation Red Edge	0.865	20
9	Water vapour	0.945	60
10	SWIR—Cirrus	1.375	60
11	SWIR	1.610	20
12	SWIR	2.190	20

### Rock sampling and analysis of Be concentrations

Representative rock samples (1 kg/sample) were collected from six beryl-bearing bedrock occurrences in south Sinai, CED, and SED of Egypt (Fig. 1), to determine the Be concentrations in the studied bedrocks. The analysis of the geochemical samples was carried out using Ultra-Trace Inductively Coupled Plasma Mass Spectrometer (ICP-MS) at Bureau Veritas Labs, Canada. A 0.25 g split was heated in an acid mixture (HNO_3_, HClO_4_, and HF) to fuming and drying, while the residue was dissolved in HCl.

### Groundwater sampling and analysis of Be and associated heavy metals

**Figure 7:**
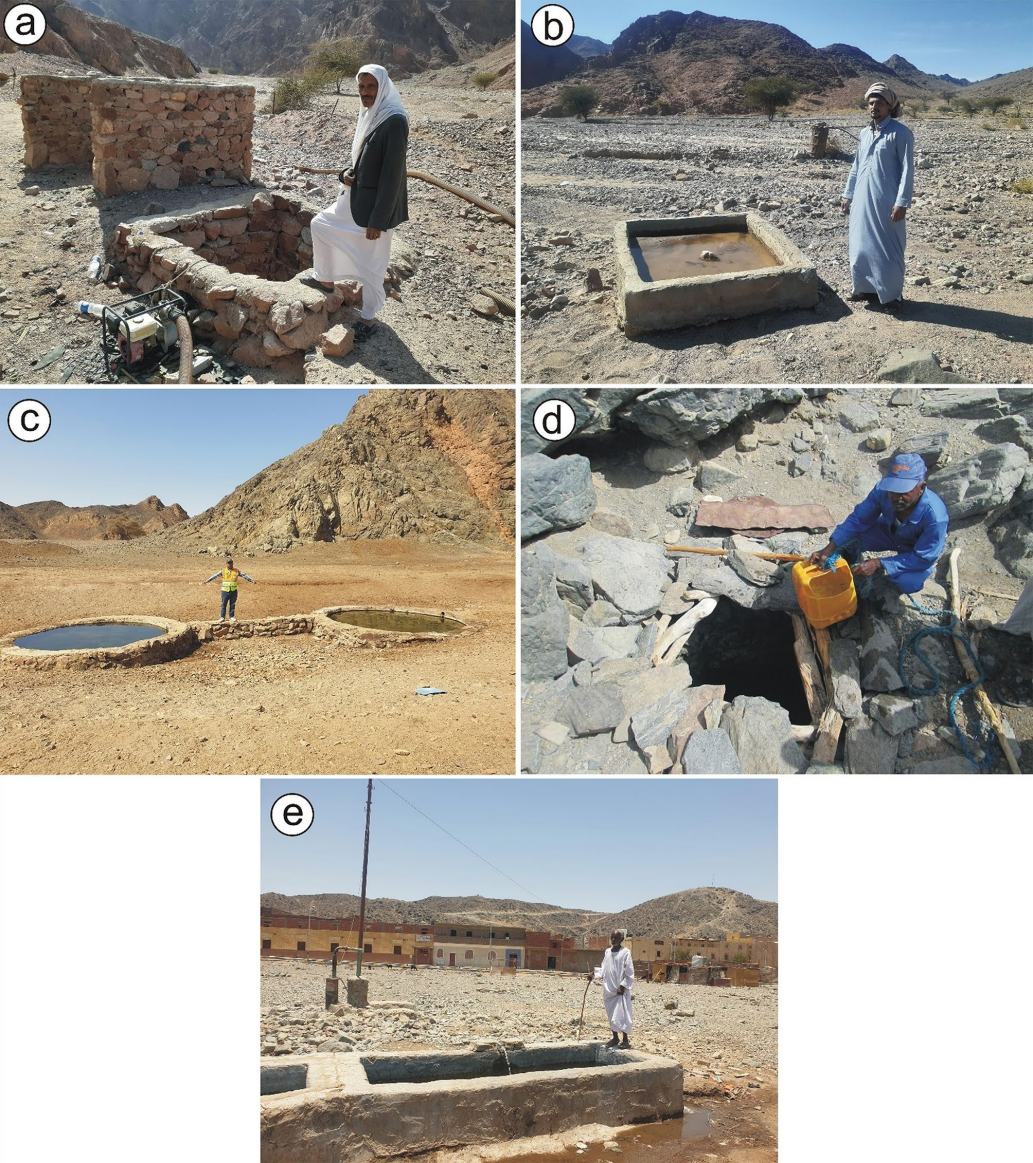
Photographs showing: (**a**) Wadi Ghazala Well, southeastern Sinai, Egypt; (**b**) Wadi Sedri Well, southwestern Sinai, Egypt; (**c**) W. Igla Well, CED, Egypt; (**d**) Um Kabu Well, Zabara-Um Addebaa belt, SED, Egypt; and (**e**) Sheikh Shazly Well in Homret Mukpid area, SED, Egypt. The human images are related to the co-author (Mohamed N. El-dawy), who is in (c), and his driver in (a), (b), (d) and (e) just as a scale.

To assess the groundwater quality parameters, the environmental risk of Be, associated heavy metals and their potential impacts on the environment and human health, a total of ten groundwater samples were collected from the main five shallow and productive groundwater wells: W. Ghazala (depth to water: 5 m; Fig. 7a) and W. Sedri (depth to water: 13 m; Fig. 7b) in South Sinai, and Igla (depth to water: 0 m; Fig. 7c), Um Kabu (depth to water: 5 m; Fig. 7d), and Shazly-Mukpid (depth to water: 18 m; Fig. 7e) in CED, and SED of Egypt. The groundwater samples were analyzed for concentrations of Be and associated major ions and heavy metals using Inductively Coupled Plasma Optical Emission Spectrometer (ICP-OES) Agilent 5100 with Synchronous Vertical Dual View (SVDV) at the National Research Centre (NRC), Giza, Egypt. Five groundwater samples were acidified with 1% HNO_3_ for Be, cations, and heavy metals analysis, while the other five unpreserved samples were used for the anions analysis. Prior to metal determination, all samples were digested according to the method^37^. For quality assurance, all groundwater samples were analyzed in triplicate and the mean values were calculated. Blank samples were also prepared during sample digestion^38^. Accuracy and precision of the metal measurements were confirmed using external reference standards from Merck Co., Germany, and National Institute of Standards and Technology (NIST), USA.

### Statistical analysis

Univariate (min, max and mean) and bivariate were applied to the investigated concentrations of Be and heavy metals in beryl-bearing rocks and nearby groundwater in this study using Statgraphics software^41^.

### Radiological risk assessment indices

The concentration of uranium (ppm), thorium (ppm), radium (ppm) and potassium (%) of the examined six areas of emerald and beryl-bearing rocks at W. Ghazala (7 samples), W. Sedri (7 samples) in south Sinai, Igla (6 samples; CED), Zabara-Um Addebaa belt (10 samples), Homret Akarem (3 samples), and Homret Mukpid (7 samples), SED of Egypt, were measured using Sodium Iodide [NaI(Tl)] scintillation detector’ with a 76 × 76 mm at Nuclear Material Authority. It is characterized by low cost, flexibility in shape, size, and linear energy. The best resolution is observed at 6% when measuring the 662 keV gamma ray from 137Cs. A cylindrical Pb shield detector with fixed bottom and cover was used to reduce γ-ray background. Pb shield includes an inner Cu cylinder (0.3 mm thick) to absorb the generated X-rays in the lead shield. Background distributions were determined using the empty specification package. The grinded samples (300–400 g) from the studied rocks were put in plastic containers, sealed well, and left for at least 21 days to accumulate free radon. The activity of ^226^Ra was estimated based on γ-lines with energies 351.9 keV (36.7%) of ^214^Pb and the 609.3 keV (46.1%), 1120.3 keV (15%), and 1764 keV (15.9%) of 214Bi. In the case of 232Th, 911.2 keV (29%), γ-lines of 228Ac and 238.6 keV (43.6%) γ-peak of ^212^Pb have been used. ^40^K activity was estimated using the 1461 keV (10.7%) γ-peak. Therefore, NaI detectors are suitable. Radiological risk parameters were estimated in the prospecting emerald and beryl-bearing rocks from six areas to infer the radiation effect on human organs. These parameters include absorbed gamma dose, outdoor and indoor annual effective dose, radium equivalent activity, internal and external indices, index of excess cancer, and effective dose to human organs.

#### Absorbed gamma-dose rate (D_air_)

This index is used to evaluate the released gamma rays more than one meter from the Earth’s surface^40,42–45^. ^226^Ra, ^232^Th, and ^40^K activity concentrations are used to estimate the D_air_ as follows:

1 $${\text{D}}_{{{\text{air}}}} \left( {{\text{nGy}}\;{\text{h}}^{{ - {1}}} } \right) = 0.{43}0\;{\text{Ra}} + 0.{666}\;{\text{Th}} + 0.0{42}\;{\text{K}}$$

where Ra, Th and K are ^226^Ra, ^232^Th, and ^40^K activity concentrations, respectively.

#### Radium equivalent activity (Ra_eq_)

Ra_eq_ is attributed to external and internal alpha particles and gamma-ray exposure dose. ^226^Ra, ^232^Th, and ^40^K activity limits are used to estimate the proper Ra_eq_ index in the examined six areas as follows^16,46,47^:

2 $${\text{Ra}}_{{{\text{eq}}}} \left( {{\text{Bq kg}}^{{ - {1}}} } \right) = {\text{Ra}} + {1}.{43}\;{\text{Th}} + 0.0{77}\;{\text{K}}$$

#### Annual effective dose (AED)

Absorbed dose (D_air_) results, conversion factor of 0.7 Sv Gy^−1^, indoor, and outdoor occupancy factors 0.8 and 0.2, respectively are used to estimate AED^48^ as follows:

3 $${\text{AED}}_{{{\text{out}}}} \left( {{\text{mSv y}}^{{ - {1}}} } \right) = {\text{D}}_{{{\text{air}}}} \left( {{\text{nGy h}}^{{ - {1}}} } \right) \times {8}.{76 }\left( {{\text{h}}/{\text{y}}} \right) \times 0.{2} \times 0.{7}\left( {{\text{Sv Gy}}^{{ - {1}}} } \right) \times {1}0^{{ - {3}}}$$

4 $${\text{AED}}_{{{\text{in}}}} \left( {{\text{mSv y}}^{{ - {1}}} } \right) = {\text{D}}_{{{\text{air}}}} \left( {{\text{nGy h}}^{{ - {1}}} } \right) \times {8}.{76}\left( {{\text{h}}/{\text{y}}} \right) \times 0.{8} \times 0.{7}\left( {{\text{Sv Gy}}^{{ - {1}}} } \right) \times {1}0^{{ - {3}}}$$

#### Internal and external radiation indices (H_in_ & H_ex_)

H_in_ & H_ex_ can be measured to infer the radiation effect on human organs. They can be estimated using the following equations^45,46^:

5 $${\text{H}}_{{{\text{in}}}} = {\text{Ra}}/{185} + {\text{Th}}/{259} + {\text{K}}/{481}0 \le {1}$$

6 $${\text{H}}_{{{\text{ex}}}} = {\text{Ra}}/{37}0 \, + {\text{Th}}/{259 } + {\text{K}}/{481}0 \le {1}$$

#### Excess lifetime cancer index (ELCR)

ELCR can be calculated using outdoor annual effective dose, over a lifetime (DL = 70 years) and cancer index (RP = 0.05 Sv) as follows:

7 $${\text{ELCR}} = {\text{AED}} \times {\text{DL}} \times {\text{RP}}$$

#### Effective dose to human organs (D_organs_)

Effective dose rate can be obtained using the below equation^49^:

8 $${\text{D}}_{{{\text{organs}}}} \left( {{\text{mSv y}}^{{ - {1}}} } \right) = {\text{AED}} \times {\text{F}}$$

Outdoor and indoor annual effective dose are AED, whereas F is the organ dose conversion factor. F values are 0.46, 0.58, 0.62, 0.64, 0.68, 0.69 and 0.82 for the Liver, Ovaries, Kidneys, Lungs, Whole Body, Testes, Bone and Marrow, respectively.

### Ethical approval

This article does not contain any studies with human participants or animals performed by any of the authors.

### Consent to participate

All authors are agreed to be as authors in the current order in this manuscript version.

## Results and discussion

### Lithological mapping of beryl-bearing bedrocks using remote sensing data

**Figure 8:**
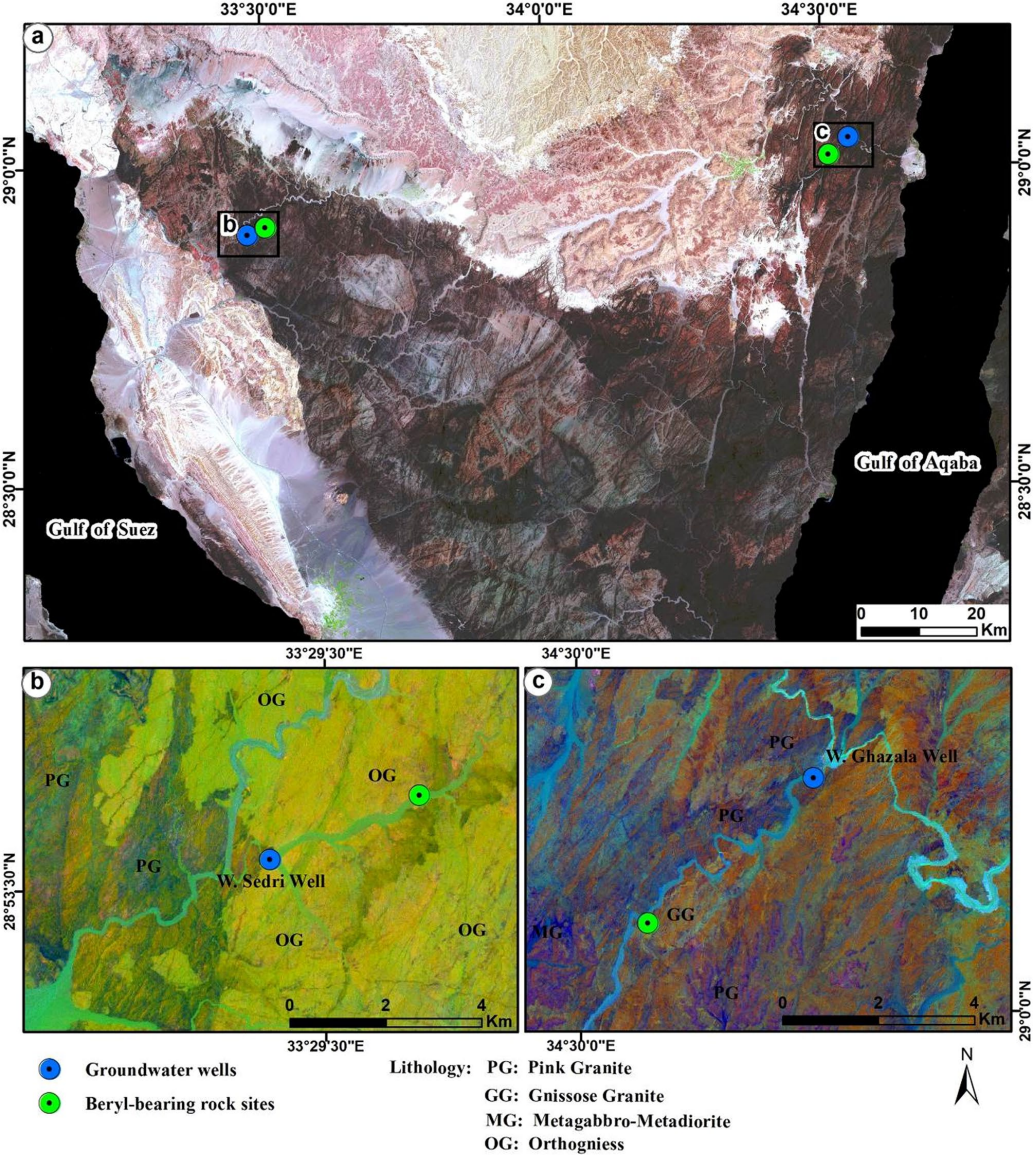
(**a**) Sentinel-2A Composite bands 8, 4, 2 showing the variable lithological rock units and locations of the investigated beryl-bearing rocks and groundwater wells in Sinai, Egypt; (**b**) Sentinel-2A PCA Composite bands 4, 3, 2 showing the distinct boundaries between beryl-bearing bedrocks in W. Sedri area, southwestern Sinai; and (**c**) W. Ghazala, southeastern Sinai, Egypt. This figure was created using software; SNAP V. 9 (source: https://step.esa.int/main/download/snap-download/) and ArcGIS V. 10.6 (source: https://www.esri.com/). Sentinel-2A data (scenes: 8; source: U.S. Geological Survey (USGS), (https://earthexplorer.usgs.gov/). The acquisition date of Sentinel-2A data is August 13, 2022, for south Sinai and August 20, 2022, for those of CED and SED of Egypt.

The Proterozoic basement rocks including beryl-bearing occurrences in south Sinai appear on the composite of Red–Green–Blue (RGB) image in Sentinel-2A false color (8, 4, 2, Fig. 8a) in dark to light red color. The sedimentary rocks (e.g., limestone) appear in rose-pink, and the Quaternary alluvial cover appears in beige to grey color, particularly in the northern and southwestern sides of the image (Fig. 8a).

Specifically, in W. Sedri, southwestern Sinai area, the Sentinel-2A PCA composite (4, 3, 2) image showed two major lithological rock units with distinctive boundaries, orthogneiss (OG), which is crossed-cut by beryl-bearing quartz veins and appears in light green to the right side, and pink granite (PG) which easily recognized by dark brownish red color in the left side of the image (Fig. 8b). In W. Ghazala, southeastern Sinai, the Sentinel-2A PCA composite (4, 3, 2) image showed three rock units, besides pink granite (PG), gneissose granite (GG), which associated with beryl mineralization, appears in orange-brown rust color, while small patches of metagabbro-metadiorite (MG) appears in a sharp blue color the left-downside of the image (Fig. 8c). The more altered soils and wadi deposits are dissected by the drainage network and show a blue-green color (Fig. 8b,c).

**Figure 9:**
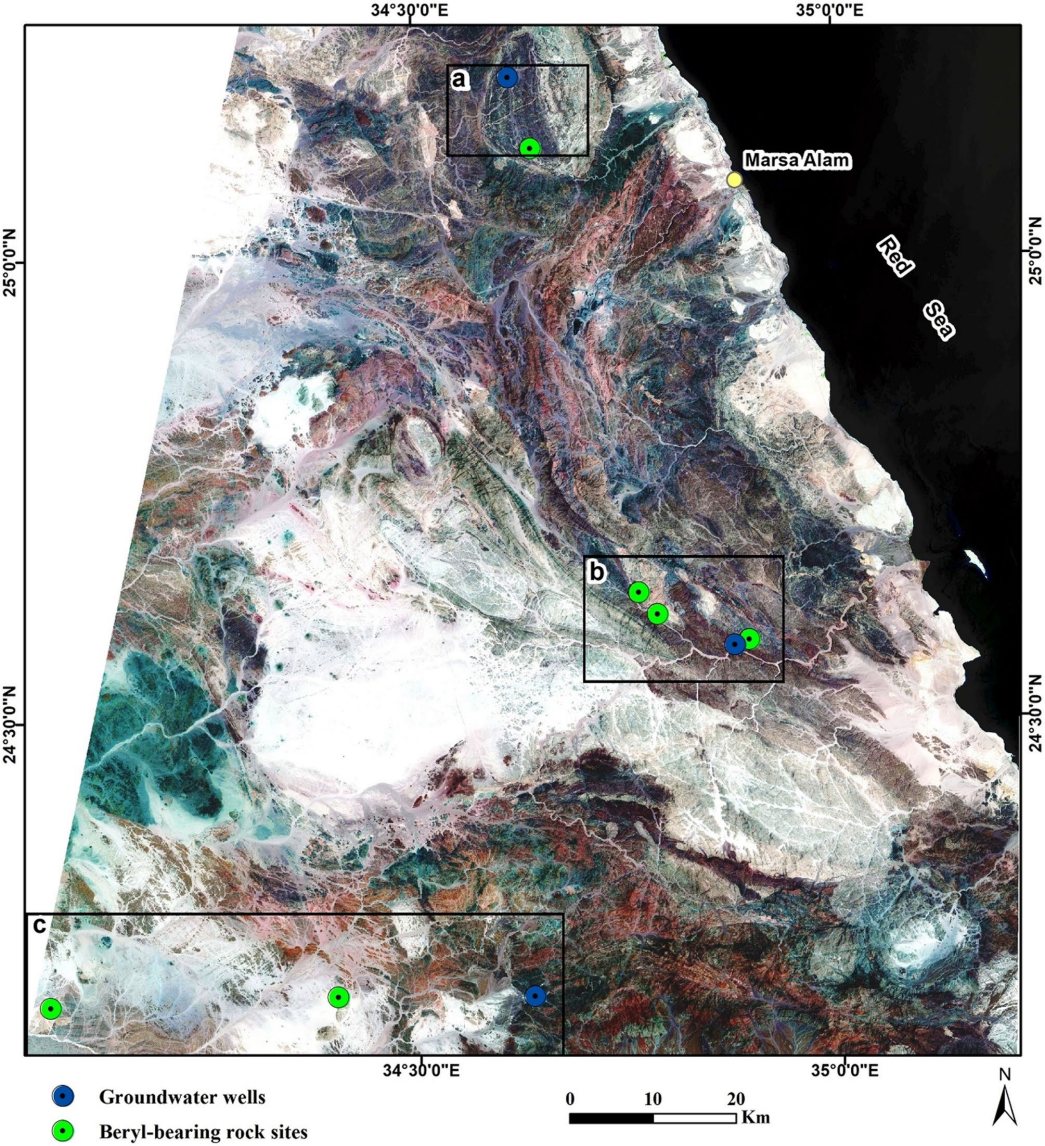
Sentinel-2A Composite bands 8, 4, 2 showing the variable lithological rock units and locations of the investigated beryl-bearing bedrocks and groundwater wells in CED, SED of Egypt. Areas covered by black boxes a, b and c are outlined in Fig. 10. This figure was created using software; SNAP V. 9 and ArcGIS V. 10.6. Sentinel-2A data (USGS), (https://earthexplorer.usgs.gov/). The acquisition date as iin Fig. 8.

**Figure 10:**
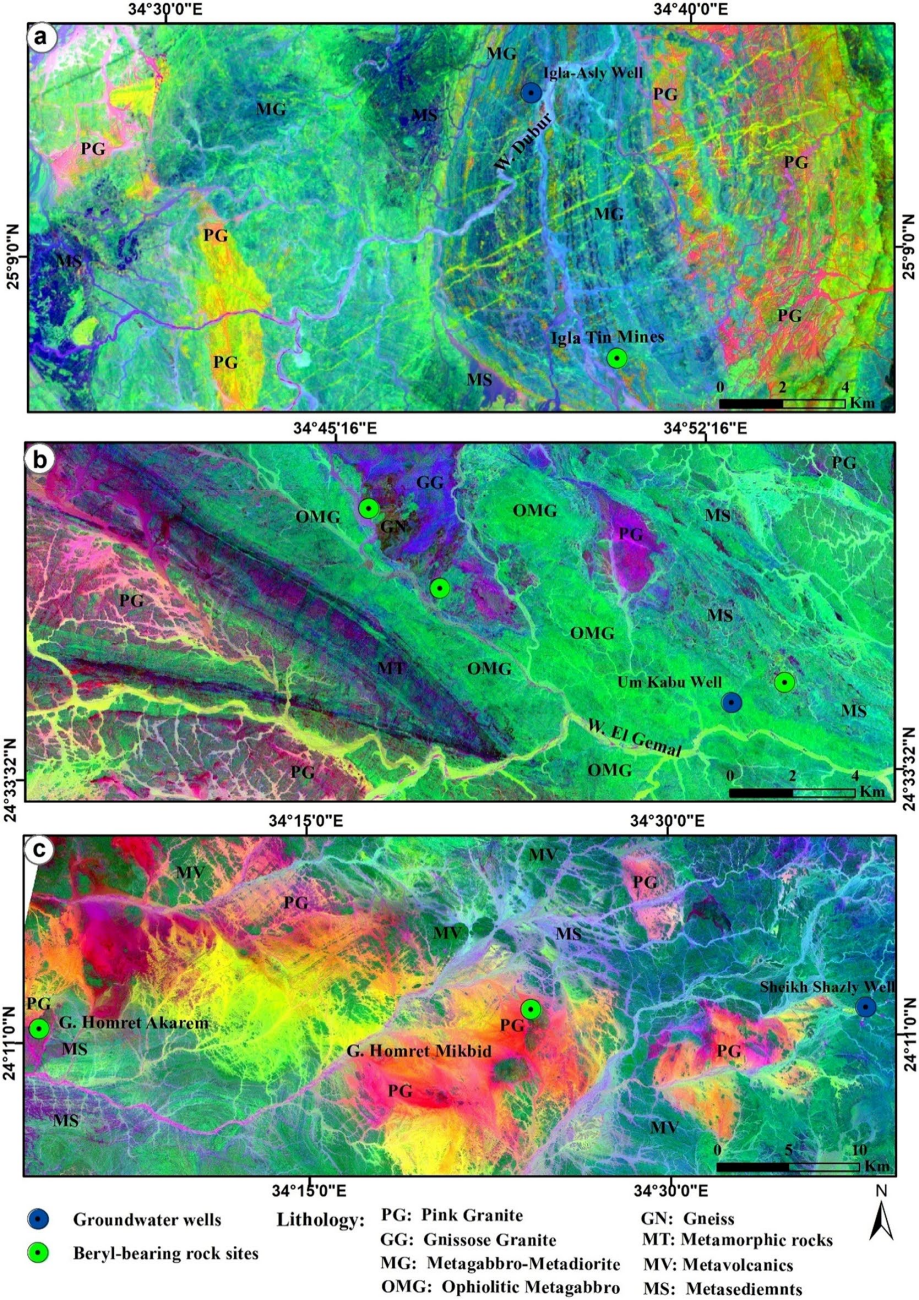
Sentinel-2A PCA Composite bands 4, 3, 2 showing the distinct boundaries between the investigated beryl-bearing bedrocks and groundwater wells in Igla mine area, CED (**a**), Zabara-Um Addebaa belt (**b**), and Homret Akarem, and Homret Mukpid areas (**c**), SED of Egypt. This figure was created using software; SNAP V. 9 and ArcGIS V. 10.6. Sentinel-2A data (USGS), (https://earthexplorer.usgs.gov/). The acquisition date as iin Fig. 8.

The Proterozoic basement rocks hosting beryl mineralization in CED, and SED of Egypt appear on the composite RGB image in Sentinel-2A false color (8, 4, 2, Fig. 9) with comparable lithological rock units and corresponding colors to south Sinai (Fig. 8a). In Igla mine area, CED, where mines of tin and other metals were common, but without any beryl occurrences, the Sentinel-2A PCA composite (4, 3, 2) image showed three rock units; pink granite (PG) with dark brownish red color, metagabbro-metadiorite (MG) appears in a light blue in the middle part of the image, and patches of metasediments (MS) with a very dark blue color near the left-downside of the image (Fig. 10a).

In Zabara-Um Addebaa belt, SED, the Sentinel-2A PCA composite (4, 3, 2) image indicated six rock units with distinctive boundaries; pink granite (PG) with dark brownish red color, ophiolitic metagabbro (OMG) appears in dark green color, metasediments (MS), where phlogopite schist contains beryl mineralization, appears in light greenish blue color, undifferentiated metamorphic rocks (MT) with a purple color, and few patches of gneissose granite (GG) with a dark blue color, and gneiss (GN) with a dark brown color (Fig. 10b). In Homret Akarem and Homret Mukpid areas, SED, the Sentinel-2A PCA composite (4, 3, 2) image indicated three rock units with distinctive boundaries; pink granite (PG) appears with brownish to pinkish red color, where beryl mineralization occurs within pegmatite veins, metasediments (MS) with light green color, and metavolcanics (MV) with dark green color (Fig. 10c). The more altered soils and wadi deposits in CED, and SED are dissected by the drainage network and show a blue-green color (Fig. 10a–c).

### Environmental risk assessment of Be concentrations in beryl-bearing bedrocks and nearby groundwater

#### Beryllium concentration in beryl-bearing bedrocks

A total of thirty-four representative rock samples, which collected from beryl-bearing bedrock occurrences in South Sinai (W. Ghazala and W. Sedri) and CED and SED of Egypt (Zabara-Um Addebaa belt, Homret Akarem, Homret Mukpid, and Igla area), (Fig. 1), were analysed for Be concentrations and reported in Table 2. Beryl in Precambrian basement rocks in Egypt has two paragenetic types: emerald in mica-schists and beryl in granitoids^6,12^. Be occurrence depends strongly on geology, thus Be is concentrated in mafic minerals and muscovite (10–50 ppm) and in plagioclase up to 20 ppm^23^. Additionally, the highest naturally occurring concentrations of Be are found in certain pegmatite bodies^53^. Abdalla^44^ reported Be concentrations with a range of 0.001–0.007% in beryl-bearing granitoids (Homret Akarem, Homret Mukpid, and Qash Amir) in the Eastern Desert of Egypt. Abdel Gawad et al.^45^ investigated the geochemistry and genesis of beryl crystals (up to 10 mm in size) from the ancient Roman mines at Wadi Nugrus, SED, Egypt. They reported that the emerald of Wadi Nugrus occurs along the contact zone between biotite schists, pegmatites, and quartz veins, and has similarities with the geological setting of the Canadian emeralds^45^. Additionally, Be reached up to 604–1700 ppm in granitic pegmatites (albite or sodic aplite dike) in W. Sikait and Wadi Nugrus areas^50^.

**Table 2:** Summary statistics of beryllium concentrations (ppm) in the investigated beryl-bearing bedrocks in south Sinai, CED, and SED of Egypt.

Occurrences		Beryl-bearing rocks	Be (ppm)			
			n	Min	Max	Mean
South Sinai	W. Ghazala	Granite	7	10	374	76
	W. Sedri	Orthogneiss	7	1	4	1.5
CED and SED of Egypt	Zabara-Um Addebaa belt	Phlogopite schist	10	8	49	19.6
	Homret Akarem	Granite	3	3	6	4.6
	Homret Mukpid	Granite	7	7	33	16.7

This study investigated Be concentrations in beryl-bearing bedrock occurrences in South Sinai, CED, and SED of Egypt. Findings showed that Be concentrations ranged from 1 to 4 ppm with an average of 1.5 ppm in orthogneiss rocks at W. Sedri, southwest Sinai, to 10–374 ppm and an average of 76 ppm in granitic rocks at W. Ghazala, southeast Sinai (Table 2). Be was not detected in the bedrocks of the Igla area, CED, while in SED, Be has a range of 8–49 ppm and an average of 19.6 ppm in phlogopite schist rocks along Zabara-Um Addebaa belt, to 3–6 ppm with an average of 4.6 ppm in granitic rocks at Homret Akarem, and up to 7–33 ppm and an average of 16.7 ppm in granitic rocks at Homret Mukpid area (Table 2).

#### Beryllium and associated heavy metals’ concentration in nearby groundwater

In such deserted areas, groundwater quality has an important role in the agricultural development and human life of the residents. The groundwater samples, collected from the main five shallow groundwater wells (W. Ghazala and W. Sedri) in south Sinai, and (Igla, Um Kabu, and Shazly-Mukpid) in CED, and SED of Egypt, were analyzed for concentrations of Be and associated major ions and heavy metals and reported in Table 3. To assess the groundwater quality parameters, the environmental risk of Be and associated heavy metals, and their potential adverse impacts on the environment and human health. The physicochemical parameters (e.g., pH, TDS, and EC) were measured in the investigated groundwater and reported in Table 3. The analyzed groundwater samples indicated slightly acidic to alkaline groundwaters (pH: 6.38–7.51; average: 6.85), medium to high salinity (TDS: 788–5350; average: 3851 mg/L), and medium (Sinai) to highly flowing groundwater in CED and SED; EC: 1.6–10.87; average: 5.81 mS/cm) (Table 3). This groundwater locates in a highly fractured basement aquifer at CED and SED, which is related to the brittle deformation associated with the Najd Fault System^51^. The studied groundwater in south Sinai, CED, and SED is more acidic (pH: 6.38–7.51) than the more alkaline groundwater (pH: 7.01–8.09) in Jordan^52^. Similarly, the flow of groundwater (EC: 1.607–10.87 mS/cm) in this study is much higher than (EC: 0.3–1.199 mS/cm) of Jordanian groundwater^38^. Additionally, high concentrations of major soluble ions were detected in the investigated groundwater (Table 3), and their average levels (mg/L) followed the decreasing order: Cl^−^ (5400) > Na^+^ (836.12) > Mg^2+^ (300.62) > Ca^2+^ (199.57) > K^+^ (22.7) > Si^4+^ (11.6) (Table 3). Overall, findings revealed that the sampled groundwater from CED and SED contained higher values of physicochemical parameters (e.g., TDS and EC) and major ions than Sinai groundwater (Table 3).

**Table 3:** Summary statistics of physicochemical parameters, major ions, beryllium and associated heavy metals in the investigated shallow groundwater in South Sinai, CED, and SED of Egypt.

		W. Ghzala	W. Sedri	Igla_Asly	Um Kabu	Shazly-Mukpid	Mean	MCL (US. EPA, 2002)
		South Sinai		CED and SED				
pH		6.58	6.85	7.51	6.92	6.38	6.85	6.5–8.5
TDS	mg/L	918	788	3440	3760	5350	3851	500
EC	mS/cm	1.874	1.607	7.04	7.67	10.87	5.81	
Cl^-^	mg/L	1412	1667	11,220	5623	7080	5400	250
Na^+^	mg/L	181	1266	201	1407	1125.6	836.12	
Mg^2+^	mg/L	27.38	16.68	33.07	181.73	41.76	300.62	
Ca^2+^	mg/L	95.3	70.83	361.14	241.93	228.68	199.57	
K^+^	mg/L	5	2.5	6	32	68	22.7	
Si^4+^	mg/L	8.6	10.15	14.23	12.7	12.3	11.6	
Be	mg/L	0.00044	0.00034	0.00016	0.00036	0.00031	0.00032	0.004
B	mg/L	0.166	0.24	1.282	0.55	0.90	0.627	1.4
Ni	mg/L	0.157	0.051	n.d	0.007	0.012	0.227	0.1
V	mg/L	0.0007	n.d	0.0415	0.431	0.041	0.514	0.1
Fe	mg/L	0.10	1. 46	0.175	0.46	0.07	0.453	0.3
Al	mg/L	0.07	0.16	0.04	0.03	0.16	0.092	0.2

n.d. means < 10 ppb.

The concentrations of the heavy metals (Be, B, Ni, V, Fe, and Al) were analyzed in the groundwater samples and reported in Table 3. Be is potentially a highly toxic element that may be mobilized especially under acidic conditions in natural waters^23^. Seepage or percolation of Be-contaminated groundwater may result in the accumulation of Be in soil and affecting the soil quality^22^. Few studies worldwide investigated Be concentrations in groundwater nearby beryl-rock mineralization. In Jordan, Be level in groundwater was 0.0006 mg/L^52^. Be level (0.00022 mg/L) was detected in neutral groundwater derived from granite rocks in southern Scotland^23^. In Germany, groundwater contained low Be levels with an average of 0.000008 mg/L^53^. A higher Be level (0.035 mg/L) was detected in the groundwater of Mexico^54,55^, while in the groundwater of Middle Russia, Be concentration was 0.009 mg/L^56^. The Ontario Ministry of Environment has set an environmental quality standard and maximum concentration level (MCL) of 0.004 mg/L for Be in groundwater^56–58^, and reported that higher Be levels could be toxic^22^.

In this study, Be concentrations in the investigated groundwater samples from south Sinai, CED, and SED of Egypt ranged from 0.00016 to 0.00044 mg/L and with an average of 0.00032 mg/L (Table 3), which is below the Be (0.004 mg/L) MCL^57,58^. One of the main sources of Be compounds in groundwater is the weathering of bedrocks (e.g., Na-feldspar and biotite granites) and soils containing Be^22,53,59^. Moreover, the highest Be concentrations occur in shallow and deep groundwaters in non-carbonate rocks^23,59^. Be levels in the investigated groundwater in this study were compared to those previously reported in groundwater around the world and followed the decreasing order: Mexico > Middle Russia > Jordan > Egypt (this study) > Scotland, UK > Germany. Most of these samples were restricted to acid groundwaters (pH < 7) and all measurable concentrations were found in non-carbonate rocks. Therefore, Be occurrence depends strongly on geology^22,52^.

The average concentrations of heavy metals associated with Be (mg/L) in the investigated groundwater, followed the decreasing order: B (0.627) > V (0.514) > Fe (0.463) > Ni (0.227) > Al (0.092) > Be (0.00032) (Table 3). Spatially, high levels of Be, and Ni were detected in the investigated groundwater from W. Ghzala, southeastern Sinai (Table 3 and Fig. 8c), while high levels of Fe and Al were recorded in groundwater of W. Sedri, southwestern Sinai (Table 3 and Fig. 8b). Additionally, high level of B was detected in groundwater of Igla-Asly well, in CED (Table 3 and Fig. 10a), and those of V was in groundwater of Um Kabu well, SED (Table 3 and Fig. 10b).

**Figure 11:**
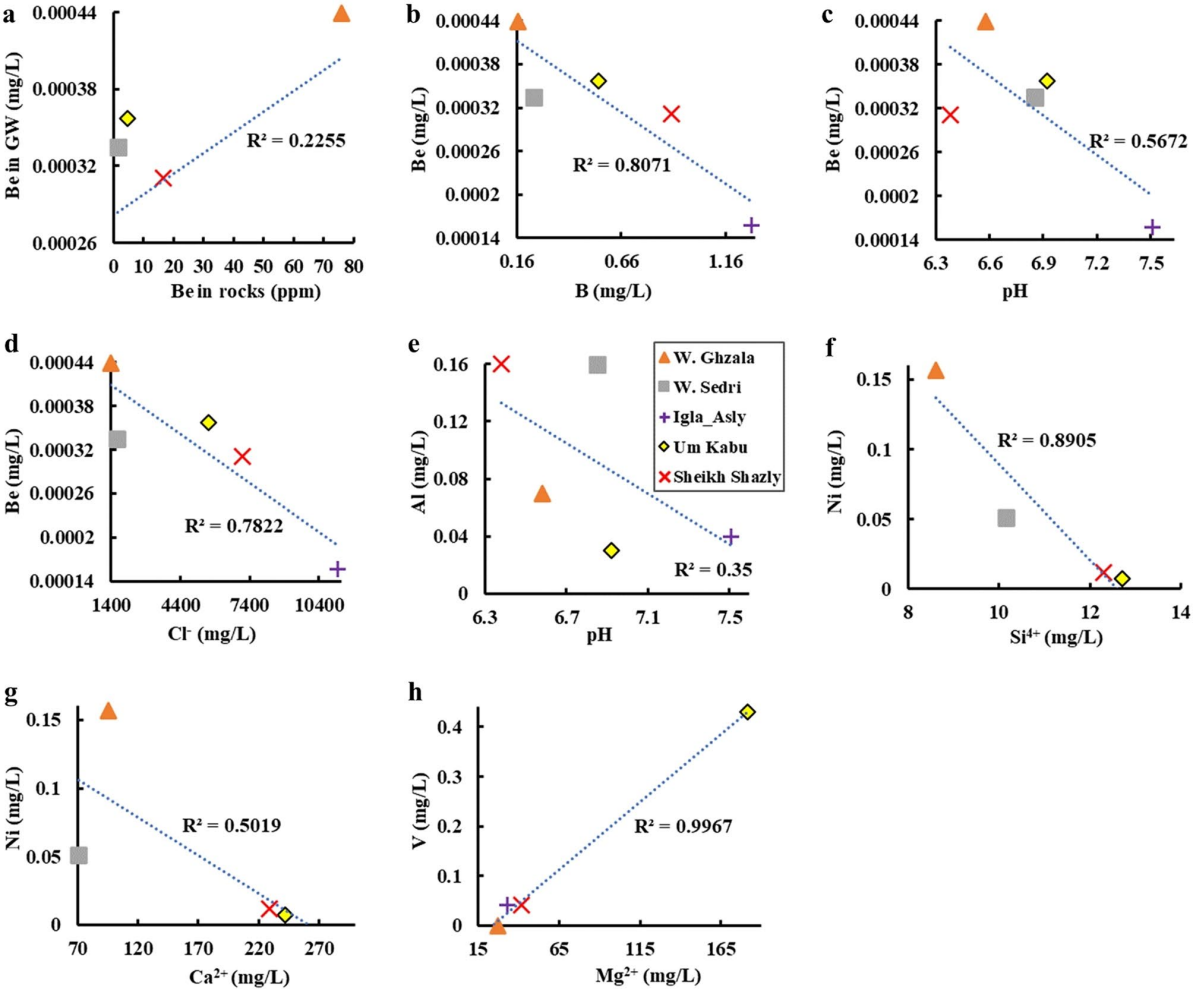
Bivariate plots show the relationship between concentrations of beryllium and associated major ions and heavy metals (mg/L) in this study. Be in groundwater versus Be in nearby rock occurrences (ppm) (**a**), B (**b**), pH (**c**) and Cl^-^ (**d**); As versus K + (**e**); Al versus pH (**f**); Ni versus Si^4+^ (**g**), and Ca^2+^ (**h**); and V versus Mg^2+^ (**i**).

The bivariate plots show the significant relationships between beryllium, pH, major ions, and associated heavy metals in the investigated groundwater of south Sinai, CED, and SED of Egypt (Fig. 11). Spatially, Be concentrations in groundwater (mg/L) were plotted versus those in beryl-bearing bedrocks (ppm) in the study area, showing that W. Ghazala, southeastern Sinai had the highest Be levels in both bedrock and groundwater, and Homret Mukpid area, SED (where Shazly well is located) showed moderate Be levels in bedrock and groundwater, while W. Sedri, southwestern Sinai, and Zabara-Um Addebaa belt (where Um Kabu well is located), SED (Fig. 10b) exhibited low Be levels in bedrocks and in groundwater as well (Fig. 11a). Noteworthy, Be concentration is unlikely to exceed 0.0002 mg/L in most acidic groundwater^23^. It is reported that zeolites could be used in normal filter beds for Be removal up to 0.0016 mg/L in groundwater^53^.

In the investigated groundwater derived from beryl-bearing bedrocks, Be was significantly and negatively correlated with B (R^2^ = 0.8; *p* < 0.05) (Fig. 10b), pH (R^2^ = 0.56; *p* < 0.05) (Fig. 11c), and Cl^−^ (R^2^ = 0.78; *p* < 0.05), (Fig. 10d). ^22,52^, reported similar results that the occurrence of Be is closely related to the low pH of the groundwater, however, ^22,52^ reported a positive correlation between Be and B in groundwater of Scotland, UK.

Some heavy metals associated with Be in groundwater showed significant relationships with pH and major ions. Al was negatively correlated with pH (R^2^ = 0.35; *p* < 0.05) (Fig. 11e) indicating that both Be and Al have similar geochemical controls^22,52^. Ni was negatively correlated with Si^4+^ (R^2^ = 0.89; *p* < 0.05) (Fig. 11f) and Ca^2+^ (R^2^ = 0.5; *p* < 0.05) (Fig. 11g). In contrast, V showed a highly significant and positive correlation with Mg^2+^ (R^2^ = 0.518; *p* < 0.05) (Fig. 11h). Overall, Fig. 11 indicates a general decrease in Be and associated heavy metals with increasing pH of groundwater, which is the usual trend with cationic metals in groundwater of Jordan^52^.

Be behavior with associated heavy metals (e.g., Al) and major ions (e.g., Mg^2+^) along with pH interval, has environmental and human health importance due to the potential intake of Be by living organisms in water^23^. Accordingly, concentrations of heavy metals associated with Be in the investigated groundwater were compared to their corresponding MCLs^57^ to assess their potential adverse impacts on the environment and human health. As a result, all B levels (0.166–1.282 mg/L) in groundwater are below (1.4 mg/L) the MCL^57^.

Overall, most levels of Be, B, Ni, Fe, Al, and V in groundwater in the investigated groundwater of south Sinai, CED, and SED are below their corresponding MCLs^53^ (Table 3; Fig. 11).

### Radiological risk assessment of beryl-bearing bedrocks

#### Radionuclide distribution

Natural radionuclide contents of the studied beryl-bearing rocks are eU (ppm), eTh (ppm), Ra (ppm) and K (%). Among the investigated areas, Zabara-Um Addebaa belt has the lowest mean values of eU (1.33 ppm) and eTh (2.1 ppm). On contrast, Homret Akarem and Homret Mukpid contain the highest eU (9 ppm) and eTh (21.57 ppm), respectively (Supplementary Table 1).

The mean values of eTh/eU of W. Ghazala (4.8), which is higher than the recommended Clark value (3.5). On the other hand, W. Sedri, Homret Mukpid, Igla, Zabara-Um Addebaa belt, Homret Akarem, and areas possess low contents of eTh/eU (2.52, 2.88, 3.22, 1.58, 1.41, respectively), which are lower than recommended Clark value (3.5). Uranium mobilization can be inferred by utilizing equation (eUm = eU − eTh/3.5). The mean calculated values are (− 1.52, − 1.31, − 4.02, − 0.95, − 0.22, − 1.05 for W. Ghazala, W. Sedri, Homret Mukpid, Igla, Zabara-Um Addebaa belt, Homret Akarem, respectively) negative, suggesting leaching out of uranium (Supplementary Table 1).

#### Activity concentrations of radionuclide

Samples measurements of W. Ghazala, W. Sedri, Homret Mukpid, Igla, Zabara-Um Addebaa belt, Homret Akarem beryl-bearing rocks, minimum, maximum, and mean of ^238^U, ^232^Th, ^226^Ra, and ^40^K activity concentrations are given in Supplementary Table 1. Radionuclides (U, Ra K, and Th, ppm) were obtained to Bq kg^−1^ to calculate their activities. Where 1 K (%) = 313 Bq kg^−1^, 1 Ra (ppm) = 11.1 Bq kg^−1^, 1 U (ppm) = 12.35 Bq kg^−1^, and 1 Th (ppm) = 4.06 Bq kg^−1^^16,47,50,51^.

The mean of ^238^U, ^232^Th, ^226^Ra and ^40^K values are 12.4, 28.2, 19 and 754 Bq kg^−1^ (respectively) for W. Ghazala, 31.90, 33.47, 33.30 and 403 Bq kg^−1^ for Sedri, 79.71, 87.15, 60.26 and 934 Bq kg^−1^ for Homret Mukpid, 6.2, 19.53, 27.55 and 508 Bq kg^−1^ for Igla, 4.13, 6.73, 24 and 2049 Bq kg^−1^ for Zabara-Um Addebaa belt and 111.6, 51.17, 85.1 and 905 Bq kg^−1^ for Homret Akarem, respectively.

Among the estimated mean ^238^U and ^232^Th, and ^226^Ra activity concentrations of the studied beryl-bearing rocks, Homret Mukpid (79, 87.15, 60.26 Bq kg^−1^) and Homret Akarem (111.6, 51.17, 85.1 Bq kg^−1^) contain the highest values, which are higher than the safety world levels^40^. This is may be attributed to their highly fractionated granitic rocks that host uranium and thorium reservoir minerals such as zircon, allanite, and monazite^16,19,21^. The ^238^U and ^232^Th activity concentrations of the examined Homret Mukpid and Homret Akarem are also higher than those of Homrit Waggat Area^16^.

On the other hand, the mean ^232^Th/^238^U values of beryl-bearing rocks in W. Ghazala, W. Sedri, Homret Mukpid, Igla, Zabara-Um Addebaa belt, Homret Akarem areas are (1.66, 1.46, 1.18, 1.14, 0.33, and 0.43, respectively, which are less than the recommended mean value^20,51^. Skewness and kurtosis are the main descriptive statistics that were performed for the studied emerald and beryl-bearing rocks in Egypt. The skewness factor is applied to the examined samples to infer asymmetric distribution. Positive and negative values of activity concentration in different areas reflect symmetric and flatness distributions, respectively. Furthermore, negative, and positive kurtosis coefficients suggest flat and preakness distribution, respectively.

#### Radiological parameters

The results of radiological assessment for the examined (six areas) beryl-bearing rocks such as absorbed gamma dose, outdoor and indoor annual effective dose, radium equivalent activity, internal and external indices, index of excess cancer, and effective dose to human organs are discussed in as follows Table 4. The mean calculated values of absorbed gamma dose rate (D_air_) are 55.87 (W. Ghazala), 53 (Sedri), 132.34 (Homret Mukpid), 37 (Igla), 120 (Homret Akarem), and 92.3 nGy h^−1^ (Zabara-Um Addebaa belt). It is noticeable that the D_air_ of Homret Mukpid, Homret Akarem, and Zabara-Um Addebaa belt is higher than the world safety (59 nGy h^−1^) level^40^.

**Table 4:** Radiological indices of beryl-bearing rocks in different areas.

	Absorbed	H_in_	H_ex_	AED_out_	AED_in_	Ra_eq_	ELCR	AGD					
	nGy h^−1^			(mSv y^−1^)	(mSv y^−1^)			μSv/y					
Ghazala (7)													
Min	38.55	0.21	0.21	0.05	0.19	73.23	0.17	0.27					
Max	76.94	0.44	0.41	0.09	0.38	160.24	0.33	0.55					
Av	55.87	0.33	0.30	0.07	0.27	117.59	0.24	0.39					
Sk	0.07	− 0.27	0.07	0.07	0.07	0.02	0.07	0.06					
Ku	− 2	0	− 2	− 2	− 2	− 2	− 2	− 2					
Sedri (7)													
Min	23.51	0.13	0.13	0.03	0.12	59.54	0.10	0.16					
Max	77.28	0.60	0.44	0.09	0.38	157.11	0.33	0.53					
Av	52.93	0.39	0.30	0.06	0.26	112.19	0.23	0.36					
Sk	− 0.37	− 0.31	− 0.37	− 0.37	− 0.37	− 0.31	− 0.37	− 0.36					
Ku	− 1.30	− 1.13	− 1.28	− 1.30	− 1.30	− 1.84	− 1.30	− 1.29					
Homrit Mukpid (7)													
Min	90.79	0.53	0.50	0.11	0.45	215.90	0.39	0.62					
Max	153.87	1.25	0.89	0.19	0.75	288.88	0.66	1.07					
Av	132.34	0.97	0.75	0.16	0.65	258.49	0.57	0.91					
Sk	− 0.88	− 0.69	− 0.84	− 0.88	− 0.88	− 0.45	− 0.88	− 0.86					
Ku	− 0.84	− 1.11	− 0.91	− 0.84	− 0.84	0.64	− 0.84	− 0.91					
Igla (6)													
Min	27.13	0.14	0.14	0.03	0.13	74.48	0.12	0.19					
Max	53.35	0.36	0.30	0.07	0.26	110.87	0.23	0.37					
Av	36.99	0.21	0.20	0.05	0.18	94.76	0.16	0.26					
Sk	0.81	1.22	0.94	0.81	0.81	− 0.24	0.81	0.77					
Ku	1.35	3.43	2.04	1.35	1.35	− 0.55	1.35	1.22					
Zabara-Um Debbaa belt (10)													
Min	87.41	0.44	0.44	0.11	0.43	178.37	0.38	0.65					
Max	102.85	0.52	0.52	0.13	0.50	213.30	0.44	0.76					
Av	92.30	0.47	0.46	0.11	0.45	191.42	0.40	0.68					
Sk	1.41	0.12	1.36	1.41	1.41	0.69	1.41	1.32					
Ku	4.26	− 1.72	4.13	4.26	4.26	0.05	4.26	3.84					
Homrit Akarem (3)													
Min	72.45	0.53	0.40	0.09	0.36	152.60	0.31	0.52					
Max	167.96	1.45	0.98	0.21	0.82	299.80	0.72	1.17					
Av	120.06	0.99	0.69	0.15	0.59	227.93	0.52	0.84					
Sk	0.01	0.00	0.00	0.01	0.01	− 0.09	0.01	0.02					
Ku	–	–	–	–	–	–	–	–					

D_organs out_ (mSv y^−1^)							D_organs in_ (mSv y^−1^)						
Liver	Ovaries	Kidneys	Lungs	Bone Marrow	Testes	Entire Body	Liver	Ovaries	Kidneys	Lungs	Bone Marrow	Testes	Entire Body
0.02	0.03	0.03	0.03	0.03	0.04	0.03	0.09	0.109	0.117	0.12	0.13	0.155	0.13
0.04	0.05	0.05	0.06	0.07	0.08	0.06	0.17	0.218	0.217	0.224	0.24	0.309	0.26
0.03	0.04	0.04	0.04	0.05	0.06	0.05	0.13	0.158	0.167	0.17	0.186	0.22	0.189
0.07	0.07	− 0.02	0.07	0.07	0.07	0.07	0.07	0.068	− 0.019	− 0.021	− 0.019	0.068	0.068
-2	− 2	− 3	− 2	− 2	− 2	− 2	− 2	− 2.29	− 2.509	− 2.55	− 2.509	− 2.29	− 2.29
0.01	0.02	0.02	0.02	0.02	0.02	0.02	0.05	0.07	0.07	0.07	0.08	0.09	0.08
0.04	0.05	0.06	0.06	0.07	0.08	0.06	0.17	0.22	0.23	0.24	0.25	0.31	0.26
0.03	0.04	0.04	0.04	0.04	0.05	0.04	0.12	0.15	0.16	0.15	0.18	0.21	0.18
-0.37	− 0.37	− 0.41	− 0.37	− 0.37	− 0.37	− 0.37	− 0.37	− 0.37	− 0.41	− 0.09	− 0.41	− 0.37	− 0.37
-1.30	− 1.30	− 1.30	− 1.30	− 1.30	− 1.30	− 1.30	− 1.30	− 1.30	− 1.30	− 2.43	− 1.30	− 1.30	− 1.30
0.05	0.06	0.07	0.07	0.08	0.09	0.08	0.20	0.26	0.28	0.29	0.31	0.37	0.31
0.09	0.11	0.12	0.12	0.13	0.15	0.13	0.35	0.44	0.47	0.48	0.52	0.62	0.52
0.07	0.09	0.10	0.10	0.11	0.13	0.11	0.30	0.38	0.41	0.42	0.45	0.53	0.45
-0.88	− 0.88	− 0.86	− 0.88	− 0.88	− 0.88	− 0.88	− 0.88	− 0.88	− 0.86	− 0.86	− 0.86	− 0.88	− 0.88
-0.84	− 0.84	− 0.87	− 0.84	− 0.84	− 0.84	− 0.84	− 0.84	− 0.84	− 0.87	− 0.96	− 0.87	− 0.84	− 0.84
0.02	0.02	0.02	0.02	0.02	0.03	0.02	0.06	0.08	0.08	0.09	0.09	0.11	0.09
0.03	0.04	0.04	0.04	0.05	0.05	0.04	0.12	0.15	0.16	0.17	0.18	0.21	0.18
0.02	0.03	0.03	0.03	0.03	0.04	0.03	0.08	0.11	0.11	0.11	0.13	0.15	0.13
0.81	0.81	0.81	0.81	0.81	0.81	0.81	0.81	0.81	0.81	0.71	0.81	0.81	0.81
1.35	1.35	1.35	1.35	1.35	1.35	1.35	1.35	1.35	1.35	0.89	1.35	1.35	1.35
0.05	0.06	0.07	0.07	0.07	0.09	0.07	0.20	0.25	0.27	0.27	0.30	0.35	0.30
0.06	0.07	0.08	0.08	0.09	0.10	0.09	0.23	0.29	0.31	0.32	0.35	0.41	0.35
0.05	0.07	0.07	0.07	0.08	0.09	0.08	0.21	0.26	0.28	0.29	0.31	0.37	0.31
1.41	1.41	1.41	1.41	1.41	1.41	1.41	1.41	1.41	1.41	1.32	1.41	1.41	1.41
4.26	4.26	4.26	4.26	4.26	4.26	4.26	4.26	4.26	4.26	3.65	4.26	4.26	4.26
0.04	0.05	0.06	0.06	0.06	0.07	0.06	0.16	0.21	0.22	0.23	0.25	0.29	0.25
0.09	0.12	0.13	0.13	0.14	0.17	0.14	0.38	0.48	0.51	0.53	0.57	0.68	0.57
0.07	0.09	0.09	0.09	0.10	0.12	0.10	0.27	0.34	0.37	0.33	0.41	0.48	0.41
0.01	0.01	0.01	0.01	0.01	0.01	0.01	0.01	0.01	0.01	0.71	0.01	0.01	0.01
–	–	–	–	–	–	–	–	–	–	–	–	–	–

**Figure 12:**
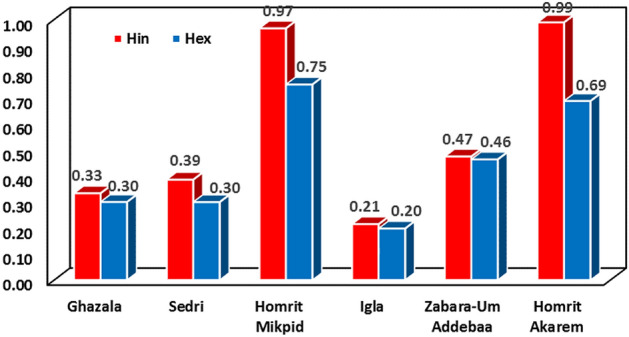
External (H_ex_) and internal (H_in_) mean values for the examined beryl-bearing rocks in W. Ghazala, W. Sedri, Homret Mukpid, Igla, Zabara-Um Addebaa belt, and Homret Akarem areas.

The mean computed values of internal hazard index (H_in_) for W. Ghazala (0.33), W. Sedri (0.39), Homret Mukpid (0.97), Igla (0.21), Zabara-Um Addebaa belt (0.47), and Homret Akarem (0.99) areas are lower than unity. Likewise, the mean external hazard (H_ex_) for W. Ghazala (0.30), W. Sedri (0.30), Homret Mukpid (0.75), Igla (0.20), Zabara-Um Addebaa belt (0.46), and Homret Akarem (0.69) areas are lower than unity (Fig. 12). This reflects that there is no significant risk associated with these rocks^16^.

The mean estimated outdoor annual effective dose (AED_out_) values are 0.07 mSv y^−1^ for W. Ghazala, 0.06 mSv y^−1^ for W. Sedri, 0.16 mSv y^−1^ for Homret Mukpid, 0.05 mSv y^−1^ for Igla, 0.11 mSv y^−1^ for Zabara-Um Addebaa belt and 0.15 mSv y^−1^ for Homret Akarem, which are comparable with the recommended level of^45^ (0.07 mSv y^−1^).

On the other hand, the mean indoor annual effective dose values (AED_in_) of W. Ghazala (0.27 mSv y^−1^), W. Sedri (0.26 mSv y^−1^), and Igla (0.18 mSv y^−1^) are less than the permissible limits of^45^, (0.41 mSv y^−1^), whereas Homret Mukpid (0.65 mSv y^−1^), Zabara-Um Addebaa belt (0.45 mSv y^−1^) and Homret Akarem (0.59 mSv y^−1^) contain the highest values. The mean Ra_eq_ of the prospecting beryl-bearing rocks in the studied areas W. Ghazala, W. Sedri, Homret Mukpid, Igla, Zabara-Um Addebaa belt, and Homret Akarem are 118, 112, 258, 95, and 191 Bq kg^−1^, respectively, which is less than the permissible safety limit (370 Bq kg^−1^) of ^45^ (Table 4). The mean ELCR data vary from 0.16 × 10^−3^ (Igla) to 0.57 × 10^−3^ (Homret Mukpid), which lies within the recommended level^63–65^.

The effective dose (D_organs_) to human organs per year is computed in Table 4. The radiation amassed in human organs for a person can be detected by D_organs_. It is noticeable that the outdoor D_organs_ data are lower than those of indoor D_organs_ and both are less than unity (recommended levels) ^62^. In addition, the indoor and outdoor (D_organs_) for liver receive the lowest dose relative to otherhuman organs. Controversy, indoor and outdoor for testes receive the highest dose^62^. Furthermore, indoor, and outdoor (D_organs_) of Homret Mukpid contain the highest values relative to other areas.

## Conclusions

Beryllium concentrations in beryl-bearing bedrocks in south Sinai (Wadi Ghazala and Wadi Sedri), CED, and SED of Egypt (Zabara-Um Addebaa belt, Homret Akarem, Homret Mukpid) were investigated in this study. The environmental risk levels of Be and associated major ions and heavy metals in shallow groundwater nearby to beryl-bearing mineralization were also evaluated. Results showed that Be concentrations ranged from 1 to 374 ppm in beryl-bearing bedrocks, while in nearby groundwater, Be has a range of 0.0001–0.00044 mg/L with an average of 0.00032 mg/L, which is within the permissible level and below (0.004 mg/L) the U.S. EPA maximum contaminant level (MCL). Considerable levels of heavy metals; Be, Ni, Fe, V, and Al were detected in the groundwater of south Sinai, while the groundwater from CED and SED of Egypt contained abundant levels of B, V, and major ions. The average levels (mg/L) of major ions in groundwater followed the order: Cl^−^ (5400) > Na^+^ (836.12) > Mg^2+^ (300.62) > Ca^2+^ (199.57) > K^+^ (22.7) > Si^4+^ (11.6). While the average of heavy metals in groundwater followed the order: B (0.627) > V (0.514) > Fe (0.463) > Ni (0.227) > Al (0.092) > Be (0.00032). The radiological risk of natural radionuclides distributed in beryl-bearing bedrocks was investigated and evaluated in this study. Among the investigated areas, Zabara-Um Addebaa belt has the lowest mean values of eU (1.33 ppm) and eTh (2.1 ppm). On contrary, Homret Akarem and Homret Mukpid contain the highest eU (9 ppm) and eTh (21.57 ppm), respectively. Radiological risk parameters (outdoor and indoor annual effective dose, radium equivalent activity, internal and external indices, index of excess cancer, and effective dose to human organs) were estimated for the studied emerald and beryl-bearing rocks to infer the radiation effect on human organs. The study findings reveal that there are no significant or adverse impacts associated with the investigated beryl-bearing rocks and nearby groundwater of south Sinai, CED and SED of Egypt.

## Supplementary Information

Supplementary Table S1.

## Data Availability

All data generated or analyzed during this study are included in this manuscript.
