# Supplementary material for: Assessing environmental and radiological impacts and lithological mapping of beryl-bearing rocks in Egypt using high-resolution sentinel-2 remote sensing images
Source: Sci Rep. 2023 Jul 17;13:11497. doi: 10.1038/s41598-023-38298-0 (PMC10352251; doi:10.1038/s41598-023-38298-0)
Supplement: Supplementary file 1 — Supplementary Table S1. [file 41598_2023_38298_MOESM1_ESM.docx]

**Assessing environmental and radiological impacts and lithological mapping of beryl-bearing rocks in Egypt using high-resolution sentinel-2 remote sensing images**

Farrage M. Khaleal^1^, Mohammed Z. El-Bialy^2^, Gehad M. Saleh^1^, El Saeed R. Lasheen^3 ⃰^, Mohamed S. Kamar^1^, Mohamed M. Omar^2^, Mohamed N. El-dawy^1^, Ahmed Abdelaal^4^

1. Nuclear Materials Authority, P.O. Box 530 El Maadi, Cairo, Egypt.
2. Geology Department, Faculty of Science, Port Said University, Port Said, Egypt
3. Geology Department, Faculty of Science, Al-Azhar University, Cairo, P.O. Box 11884, Egypt
4. Environmental Sciences Department, Faculty of Science, Port Said University, Port Said, 42522 Egypt

*Corresponding author: El Saeed R. Lasheen; [elsaeedlasheen@azhar.edu.eg](mailto:elsaeedlasheen@azhar.edu.eg)

ORCID: 0000-0002-7931-5421

**Table S1:** Radionuclide distribution in beryl-bearing rocks and their activity concentrations.

| **Areas/No of samples** | **eU** | **eTh** | **Ra** | **K** | **Um** | **^238^U** | **^226^Th** | **^226^Ra** | **^40^K** |  |
| --- | --- | --- | --- | --- | --- | --- | --- | --- | --- | --- |
|  | **(ppm)** | **(ppm)** | **(ppm)** | **(%)** |  | **Bq kg^-1^** | **Bq kg^-1^** | **Bq kg^-1^** | **Bq kg^-1^** |  |
| **Ghazala (7)** | | | | | | | | | | |
| **Min** | 1 | 6.00 | 1.00 | 0.90 | -8.00 | 0.00 | 24.24 | 11.10 | 281.70 |  |
| **Max** | 2 | 8.00 | 2.00 | 3.81 | -4.00 | 24.80 | 32.32 | 22.20 | 1192.53 |  |
| **Av.** | 1.4 | 6.71 | 1.71 | 2.34 | -5.71 | 12.40 | 28.28 | 19.03 | 754.78 |  |
| **Sk** | 0.4082483 | 0.46 | -0.95 | 0.21 | -0.55 | 0.00 | 0.00 | -0.95 | -0.05 |  |
| **Ku** | -3.333333 | 0 | -1 | -1 | 0 | -1 | -1 | -1 | -2 |  |
| **Sedri (7)** | | | | | | | | | | |
| **Min** | 1.00 | 2.00 | 2.00 | 0.97 | -8.00 | 0.00 | 8.08 | 22.20 | 303.61 |  |
| **Max** | 5.00 | 12.00 | 4.00 | 1.80 | -1.00 | 62.00 | 48.48 | 44.40 | 563.40 |  |
| **Av.** | 3.00 | 7.57 | 2.86 | 1.26 | -5.00 | 31.89 | 33.47 | 33.30 | 402.88 |  |
| **Sk** | 0.00 | -0.34 | 0.27 | 1.25 | 0.34 | -0.13 | -0.60 | 0.00 | 0.73 |  |
| **Ku** | -0.30 | -0.80 | -1.82 | 3.55 | -0.91 | -0.64 | -0.91 | -2.60 | -1.09 |  |
| **Homrit Mukpid (7)** | | | | | | | | | | |
| **Min** | 2.00 | 16.00 | 5.00 | 2.65 | -23.00 | 0.00 | 64.64 | 55.50 | 829.45 |  |
| **Max** | 11.00 | 24.00 | 6.00 | 3.62 | -9.00 | 136.40 | 96.96 | 66.60 | 1086.11 |  |
| **Av.** | 7.50 | 21.57 | 5.43 | 3.12 | -15.14 | 79.71 | 88.30 | 60.26 | 934.53 |  |
| **Sk** | -0.68 | -1.32 | 0.29 | -0.02 | -0.58 | -0.50 | -1.34 | 0.29 | 0.43 |  |
| **Ku** | 0.56 | 2.78 | -2.80 | -2.19 | 2.07 | -0.96 | 2.63 | -2.80 | -1.93 |  |
| **Igla (6)** | | | | | | | | | | |
| **Min** | 1.00 | 3.00 | 2.00 | 1.37 | -6.00 | 0.00 | 12.12 | 22.20 | 453.85 |  |
| **Max** | 2.00 | 8.00 | 3.00 | 2.02 | -2.00 | 24.80 | 32.32 | 33.30 | 632.26 |  |
| **Av.** | 1.50 | 4.83 | 2.50 | 1.61 | -4.33 | 6.20 | 19.53 | 27.75 | 507.58 |  |
| **Sk** | - | 0.61 | 0.00 | 0.94 | 0.28 | 1.12 | 0.61 | 0.00 | 1.15 |  |
| **Ku** | - | -0.06 | -3.33 | 1.93 | -1.48 | 1.43 | -0.06 | -3.33 | 2.58 |  |
| **Zabara-Um Debbaa belt (10)** | | | | | | | | | | |
| **Min** | 1.00 | 1.00 | 2.00 | 5.67 | -3.00 | 0.00 | 4.04 | 22.20 | 1953.12 |  |
| **Max** | 1.00 | 3.00 | 3.00 | 7.21 | 0.00 | 12.40 | 12.12 | 33.30 | 2256.73 |  |
| **Av.** | 1.00 | 1.67 | 2.17 | 6.45 | -1.33 | 4.13 | 6.73 | 24.05 | 2048.59 |  |
| **Sk** | - | 0.63 | 1.79 | 0.00 | -0.05 | 0.71 | 0.63 | 1.79 | 0.93 |  |
| **Ku** | - | -0.30 | 6.00 | 0.48 | -1.55 | -1.88 | -0.30 | 6.00 | 0.54 |  |
| **Homrit Akarem (3)** | | | | | | | | | | |
| **Min** | 4.00 | 4.00 | 5.00 | 2.45 | -7.00 | 49.60 | 16.16 | 55.50 | 876.40 |  |
| **Max** | 14.00 | 21.00 | 10.00 | 3.07 | 0.00 | 173.60 | 84.84 | 111.00 | 960.91 |  |
| **Av.** | 9.00 | 12.67 | 7.67 | 2.77 | -3.67 | 111.60 | 51.17 | 85.10 | 904.57 |  |
| **Sk** | 0.00 | -0.07 | -0.24 | -0.16 | 0.17 | 0.00 | -0.07 | -0.24 | 0.71 |  |
| **Ku** | - | - | - | - | - | - | - | - | - |  |

Um= Uranium mobilization
